# Comment on Lopes et al. Adiposity Metabolic Consequences for Adolescent Bone Health. *Nutrients* 2022, *14*, 3260

**DOI:** 10.3390/nu15234952

**Published:** 2023-11-29

**Authors:** Barbara Joan Boucher

**Affiliations:** The Blizard Institute, School of Medicine & Dentistry, Queen Mary University of London, London E1 2AT, UK; bboucher@doctors.org.uk

This interesting report [1] examines the problems linking childhood and adolescent obesity to reductions in bone health, indicating that the major risk factors for poor bone health in young people with obesity were various nutrient deficiencies, especially in the intakes of calcium and vitamin D, sedentary lifestyles and reduced sun exposure, together with various epigenetic changes affecting bone health both before and after birth. However, it is of interest that a maternal lack of vitamin D is causal for later life obesity in human offspring, likely due to specific epigenetic defects resulting from maternal D deficiency [2]. In adults of all age groups, however, a bi-directional Mendelian randomisation analysis of data from 21 adult cohorts demonstrated that vitamin D deficiency is not causal for obesity; rather, obesity is causal for vitamin D deficiency [3]. This effect of obesity is unlikely to be solely due to the postulated increase in the dilution of available 25(OH)D in enlarged fat masses, as the activity of the hepatic 25-hydroxlase enzyme producing 25(OH)D has been found to be reduced in obesity [4]. Together, these data mean that the avoidance of D deficiency in pregnancy is especially important for later bone health and that the 1^1/2^-to-3-fold larger intakes of vitamin D needed by overweight and obese subjects, respectively, to achieve a vitamin D status adequate for the support of bone health [5] should be routinely achieved both in pregnant women and in the overweight and obese; this must be achieved throughout one’s life span for the protection of bone health, as well as various other health benefits reportedly associated with adequate vitamin D status [6].
